# Periodic solutions in next generation neural field models

**DOI:** 10.1007/s00422-023-00969-6

**Published:** 2023-08-03

**Authors:** Carlo R. Laing, Oleh E. Omel’chenko

**Affiliations:** 1 School of Mathematical and Computational Sciences, Massey University, Private Bag 102-904 NSMC, Auckland, New Zealand; 2 Institute of Physics and Astronomy, University of Potsdam, Karl-Liebknecht-Str. 24/25, 14476 Potsdam, Germany

**Keywords:** Neural field model, Riccati equation, Theta neuron, Ott/Antonsen, Self-consistency

## Abstract

We consider a next generation neural field model which describes the dynamics of a network of theta neurons on a ring. For some parameters the network supports stable time-periodic solutions. Using the fact that the dynamics at each spatial location are described by a complex-valued Riccati equation we derive a self-consistency equation that such periodic solutions must satisfy. We determine the stability of these solutions, and present numerical results to illustrate the usefulness of this technique. The generality of this approach is demonstrated through its application to several other systems involving delays, two-population architecture and networks of Winfree oscillators.

## Introduction

The collective behaviour of large networks of neurons is a topic of ongoing interest. One of the simplest forms of behaviour is a periodic oscillation, which manifests itself as a macroscopic rhythm created by the synchronous firing of many neurons. Such oscillations have relevance to rhythmic movement (Lindén et al. 2022), epilepsy (Jiruska et al. 2013; Netoff and Schiff 2002), schizophrenia (Uhlhaas and Singer 2010) neural communication (Reyner-Parra and Huguet 2022) and EEG/MEG modelling (Byrne et al. 2022), among others. In different networks, oscillations may arise from mechanisms such as synaptic delays (Devalle et al. 2018), the interaction of excitatory and inhibitory populations (Börgers and Kopell 2005; Schmidt and Avitabile 2020), having sufficient connectivity in an inhibitory network (di Volo and Torcini 2018), or the finite width of synaptic pulses emitted by neurons (Ratas and Pyragas 2016). The modelling and simulation of such networks is essential in order to investigate their dynamics.

Among the many types of model neurons used when studying networks of neurons, theta neurons (Ermentrout and Kopell 1986), Winfree oscillators (Ariaratnam and Strogatz 2001) and quadratic integrate-and-fire (QIF) neurons (Latham et al. 2000) are some of the simplest. These three types of model neurons have the advantage that their mathematical form often allows infinite networks of such neurons to be analysed exactly using the Ott-Antonsen method (Ott and Antonsen 2008, 2009). We continue along those lines in this paper.

Here we largely consider a spatially-extended network of neurons, in which the neurons can be thought of as lying on a ring. Such ring networks have been studied in connection with modelling head direction (Zhang 1996) and working memory (Funahashi et al. 1989; Wimmer et al. 2014), for example, and been studied by others (Esnaola-Acebes et al. 2017; Laing and Chow 2001; Kilpatrick and Ermentrout 2013). We consider a network of theta neurons. The theta neuron is a minimal model for a neuron which undergoes a saddle-node-on-invariant-circle (SNIC) bifurcation as a parameter is varied (Ermentrout and Kopell 1986). The theta neuron is exactly equivalent to a quadratic integrate-and-fire (QIF) neuron, under the assumption of infinite firing threshold and reset values (Montbrió et al. 2015; Avitabile et al. 2022; Devalle et al. 2017). The coupling in the network is nonlocal synaptic coupling, implemented using a spatial convolution with a translationally-invariant coupling kernel.

We studied such a model in the past (Omel’chenko and Laing 2022), concentrating on describing spatially-uniform states and also stationary “bump” states in which there is a spatially-localised group of active neurons while the remainder of the network is quiescent. We determined the existence and stability of such states and also found regions of parameter space in which neither of these types of states were stable. Instead, we sometimes found solutions which were periodic in time. In this paper we study such periodic solutions using a recently-developed technique (Omel’chenko 2023, 2022) which is significantly faster than the standard approach.

This technique was successfully applied to describe traveling and breathing chimera states in nonlocally coupled phase oscillators (Omel’chenko 2023, 2022). In this paper, we generalize this technique to neural models and illustrate its possibilities with several examples. We start with a ring network of theta neurons and consider periodic bump states there. We describe a continuation algorithm, perform linear stability analysis of such states and derive some useful formulas, for example, for average firing rates. We show that the same approach works in the presence of delays and for two-population models. Finally, we show that Winfree oscillators are also treatable by the proposed technique.

The structure of the paper is as follows. In Sect. 1 we present the discrete network model and its continuum-level description. The bulk of the paper is in Sect. 2, where we show how to describe periodic states in a self-consistent way, and show numerical results from implementing our algorithms. Other models are considered in Sect. 3 and we end in Sect. 4. The Appendix contains useful results regarding the complex Riccati equation.

## Theta neuron network model

The model we consider first is that in Omel’chenko and Laing (2022), Laing (2014a) and Laing and Omel’chenko (2020), which we briefly present here. The discrete network consists of *N* synaptically coupled theta neurons described by

1 $$\begin{aligned}{} & {} \frac{\displaystyle \textrm{d}\theta _j}{\displaystyle \textrm{d}t} = 1 - \cos \theta _j + ( 1 + \cos \theta _j ) ( \eta _j + \kappa I_j ),\nonumber \\{} & {} \quad j=1,\ldots ,N, \end{aligned}$$

where each $\theta _j\in [0,2\pi ]$ is an angular variable. The constant $\kappa$ is the overall strength of coupling within the network, and the current entering the *j*th neuron is $\kappa I_j$ where

2 $$\begin{aligned} I_j(t) = \frac{\displaystyle 2\pi }{\displaystyle N} \sum \limits _{k=1}^N K_{jk} P_n(\theta _k(t)) \end{aligned}$$

where

$$\begin{aligned} P_n(\theta ) = a_n ( 1 - \cos \theta )^n \end{aligned}$$

is a pulsatile function with a maximum at $\theta =\pi$ (when the neuron fires) and $a_n$ is chosen according to the normalization condition

$$\begin{aligned} \int _0^{2\pi } P_n(\theta ) d\theta = 2\pi . \end{aligned}$$

Increasing *n* makes $P_n(\theta )$ “sharper” and more pulse-like. The excitability parameters $\eta _j$ are chosen from a Lorentzian distribution with mean $\eta _0$ and width $\gamma > 0$

$$\begin{aligned} g(\eta ) = \frac{\displaystyle \gamma }{\displaystyle \pi } \frac{\displaystyle 1}{\displaystyle ( \eta - \eta _0 )^2 + \gamma ^2}. \end{aligned}$$

The connectivity within the network is given by the weights $K_{jk}$ which are defined by $K_{jk}=K(2\pi (j-k)/N)$ where the coupling kernel is

3 $$\begin{aligned} K(x) = \frac{\displaystyle 1}{\displaystyle 2\pi } ( 1 + A \cos x ) \end{aligned}$$

for some constant *A*. Note that the form of coupling implies that the neurons are equally-spaced around a ring, with periodic boundary conditions. Such a network can support solutions which are — at a macroscopic level — periodic in time; see Fig. 3 in Omel’chenko and Laing (2022). Such solutions are unlikely to be true periodic solutions of (1), since for a typical realisation of the $\eta _j$, one or more neurons will have extreme values of this parameter, resulting in them not frequency-locking to other neurons.

Note that the model we study here has only one neuron at each spatial position, and for $A>0$ the connections between nearby neurons are more positive than those between distant neurons. For $A>1$ neurons on opposite sides of the domain inhibit one another, as the connections between them are negative, giving a “Mexican-hat” connectivity. For $A<0$ connections between neurons on opposite sides of the domain are more positive than those between nearby neurons. Such a model with one population of neurons and connections of mixed sign can be thought of as an approximation of a network with populations of both excitatory and inhibitory neurons (Pinto and Ermentrout 2001; Esnaola-Acebes et al. 2017).

Using the Ott/Antonsen ansatz (Ott and Antonsen 2008, 2009), one can show that in the limit $N\rightarrow \infty$, the long-term dynamics of the network (1) can be described by

4 $$\begin{aligned} \frac{\displaystyle \partial z}{\displaystyle \partial t}= & {} \frac{\displaystyle (i \eta _0 - \gamma ) ( 1 + z )^2 - i ( 1- z )^2 }{\displaystyle 2} \nonumber \\{} & {} + \kappa \frac{\displaystyle i ( 1 + z )^2 }{\displaystyle 2} {\mathcal {K}} H_n(z), \end{aligned}$$

where

5 $$\begin{aligned} ({\mathcal {K}} \varphi ) (x) = \int _0^{2\pi } K(x - y) \varphi (y) dy \end{aligned}$$

is the convolution of *K* and $\varphi$ and

$$\begin{aligned} H_n(z) = a_n \left[ C_0 + \sum \limits _{q=1}^n C_q \left( z^q + {\overline{z}}^q \right) \right] , \end{aligned}$$

where

$$\begin{aligned} C_q = \sum \limits _{k=0}^n \sum \limits _{m=0}^k \frac{\displaystyle \delta _{k-2m,q} (-1)^k n!}{\displaystyle 2^k (n - k)! m! (k - m)!}. \end{aligned}$$

For our computations we set $n=2$, so that

$$\begin{aligned} H_2(z) =(2/3)[3/2-(z+\bar{z})+(z^2+\bar{z}^2)/4] \end{aligned}$$

where overline indicates the complex conjugate. Periodic solutions like those studied below were also found with $n=5$, for example, so the choice of *n* is not critical. The wider question of the effects of pulse shape and duration is an interesting one (Pietras 2023).

Equation (4) is an integro-differential equation for a complex-valued function *z*(*x*, *t*), where $x\in [0,2\pi ]$ is position on the ring. *z*(*x*, *t*) is a local order parameter and can be thought of as the average of $e^{i\theta }$ for neurons in a small neighbourhood of position *x*. The magnitude of *z* is a measure of how synchronised the neurons are, whereas its argument gives the most likely value of $\theta$ (Laing 2014a). Using the equivalence of theta and QIF neurons, one can also provide a relevant biological interpretation of *z*. Namely, defining $W \equiv (1 - {\overline{z}})/(1 + {\overline{z}})$, one can show (Laing 2015; Montbrió et al. 2015) that $\pi ^{-1} \textrm{Re}\,W$ is the flux through $\theta = \pi$ or the instantaneous firing rate of neurons at position *x* and time *t*. Similarly, if $V_j = \tan (\theta _j/2)$ is the voltage of the *j*th QIF neuron, the mean voltage at position *x* and time *t* is given by $\textrm{Im}\,W$. Note that physically relevant solutions of Eq. (4) must assume values $|z|\le 1$. In other words, we are interested only in solutions $z\in \overline{{\mathbb {D}}}$, where

$$\begin{aligned} {\mathbb {D}} = \{ z\in {\mathbb {C}}: |z| < 1 \} \end{aligned}$$

is the unit disc in the complex plane.

Equations of the form (4)–(5) are sometimes referred to as *next generation neural field models* (Byrne et al. 2019; Coombes and Byrne 2019) as they have the form of a neural field model (an integro-differential equation for a macroscopic quantity such as “activity” (Laing et al. 2002; Amari 1977)) but are derived exactly from a network like (1), rather than being of a phenomenological nature.

## Periodic states

**Fig. 1:**
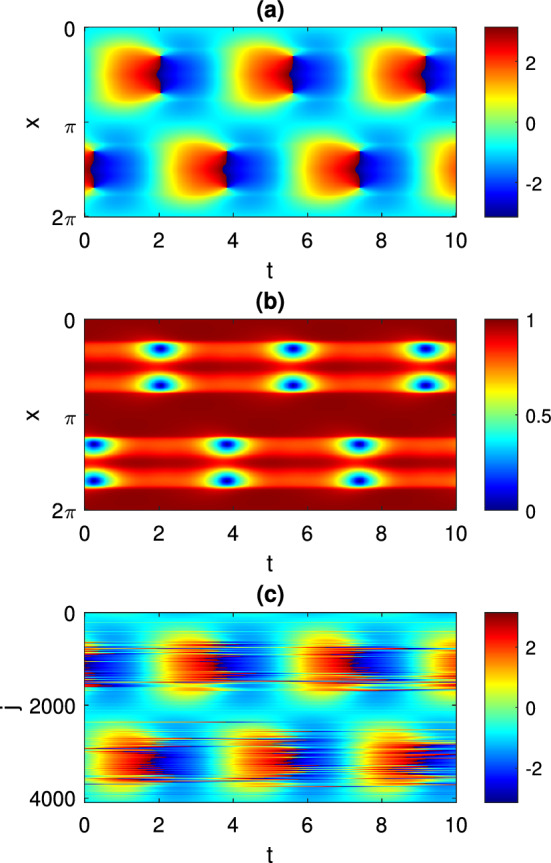
A typical periodic solution of Eq. (4). **a** $\arg {(z(x,t))}$. **b** |*z*(*x*, *t*)|. **c** A realization of this solution in a network of $N = 4096$ theta neurons described by Eq. (1). $\theta _j$ is shown in color. Parameters: $A=-5$, $\eta _0=-0.7$, $\kappa =1$, $\gamma =0.01$

In this paper we focus on states with periodically oscillating macroscopic dynamics. For the mean field Eq. (4) these correspond to periodic solutions

$$\begin{aligned} z = a(x,t) \end{aligned}$$

where $a(x,t+T) = a(x,t)$ for some $T > 0$. The frequency corresponding to *T* is denoted by $\omega = 2\pi / T$. An example of a periodic solution is shown in Fig. 1, panels (a) and (b). Figure 1c shows a realization of the same solution in the discrete network (1). We note that this solution has a spatio-temporal symmetry: it is invariant under a time shift of half a period followed by a reflection about $x=\pi$. However, we do not make use of this symmetry in the following calculations.

The appearance of periodic solutions in this model is in contrast with classical one-population neural field models for which they do not seem to occur (Laing and Troy 2003). This is another example of next generation neural field models showing more complex time-dependent behaviour than classical ones (Laing and Omel’chenko 2020).

A straight-forward way to study a periodic orbit like that in Fig. 1 would be discretise Eq. (4) on a spatially-uniform grid and approximate the convolution using matrix/vector multiplication or otherwise, resulting in a large set of coupled ordinary differential equations. The periodic solution of these could then be studied using standard techniques (Laing 2014a), but note that the computational complexity of this would typically scale as $\sim N^2$, where *N* is the number of spatial points used in the grid. Instead we propose here an alternative method based on the ideas from Omel’chenko (2023, 2022), which allows us to perform the same calculations with only $\sim N$ operations. The main ingredients of this method are explained in Sects. 2.1 and 2.2. They include the description of the properties of complex Riccati equation and the derivation of the self-consistency equation for periodic solutions of Eq. (4). In Sect. 2.3 we explain how the self-consistency equation can be solved in the case of coupling function (3). Then in Sect. 2.4 we report some numerical results obtained with our method. In addition, in Sect. 2.5 we perform a rigorous linear stability analysis of periodic solutions of Eq. (4) by considering the spectrum of the corresponding monodromy operator. Finally, in Sect. 2.6, we show how the mean field Eq. (4) can be used to predict the average firing rate distribution in the neural network (1).

### Periodic complex Riccati equation and Möbius transformation

By the time rescaling $u(x,t) = z(x,t/\omega )$ we can rewrite Eq. (4) in the form

6 $$\begin{aligned} \omega \frac{\displaystyle \partial u}{\displaystyle \partial t}= & {} \frac{\displaystyle (i \eta _0 - \gamma ) ( 1 + u )^2 - i ( 1- u )^2 }{\displaystyle 2} \nonumber \\{} & {} + \kappa \frac{\displaystyle i ( 1 + u )^2 }{\displaystyle 2} {\mathcal {K}} H_n(u), \end{aligned}$$

such that the above *T*-periodic solution of Eq. (4) corresponds to a $2\pi$-periodic solution of Eq. (6). Then, dividing (6) by $\omega$ and reordering the terms we can see that Eq. (6) is equivalent to a complex Riccati equation

7 $$\begin{aligned} \frac{\displaystyle \partial u}{\displaystyle \partial t}= & {} i \left( W(x,t) + \zeta - \frac{\displaystyle 1}{\displaystyle 2\omega } \right) + 2 i \left( W(x,t) + \zeta + \frac{\displaystyle 1}{\displaystyle 2\omega } \right) u \nonumber \\{} & {} + i \left( W(x,t) + \zeta - \frac{\displaystyle 1}{\displaystyle 2\omega } \right) u^2, \end{aligned}$$

with the $2\pi$-periodic in *t* coefficient

8 $$\begin{aligned} W(x,t) = \frac{\displaystyle \kappa }{\displaystyle 2\omega } {\mathcal {K}} H_n(u) \end{aligned}$$

and

9 $$\begin{aligned} \zeta = \frac{\displaystyle \eta _0 + i \gamma }{\displaystyle 2\omega }. \end{aligned}$$

In Omel’chenko (2023) it was shown (see also Proposition 2 and Remark 2 in the Appendix for additional details) that independent of the choice of the real-valued periodic function *W*(*x*, *t*), parameters $\zeta \in {\mathbb {C}}_{\textrm{up}}=\{ z\in {\mathbb {C}}: \textrm{Im}\,z > 0\}$ and $\omega > 0$, for every fixed $x\in [0,2\pi ]$ Eq. (7) has a unique stable $2\pi$-periodic solution *U*(*x*, *t*) that lies entirely in the open unit disc ${\mathbb {D}}$. Denoting the corresponding solution operator by

$$\begin{aligned} {\mathcal {U}}: C_{\textrm{per}}([0,2\pi ];{\mathbb {R}})\times {\mathbb {C}}_{\textrm{up}}\times (0,\infty )\rightarrow C_{\textrm{per}}([0,2\pi ];{\mathbb {D}}), \end{aligned}$$

we can write the $2\pi$-periodic solution of interest as

10 $$\begin{aligned} U(x,t) = {\mathcal {U}}\left( W(x,t), \frac{\displaystyle \eta _0 + i \gamma }{\displaystyle 2\omega }, \omega \right) . \end{aligned}$$

Note that $C_{\textrm{per}}([0,2\pi ];{\mathbb {R}})$ here denotes the space of all real-valued continuous $2\pi$-periodic functions, while the notation $C_{\textrm{per}}([0,2\pi ];{\mathbb {D}})$ stands for the space of all complex continuous $2\pi$-periodic functions with values in the open unit disc ${\mathbb {D}}$. Importantly, the variable *x* appears in formula (10) as a parameter so that the function $W(x,\cdot )\in C_{\textrm{per}}([0,2\pi ];{\mathbb {R}})$ with a fixed *x* is considered as the first argument of the operator ${\mathcal {U}}$.

As for the operator ${\mathcal {U}}$, although it is not explicitly given, its value can be calculated without resource-demanding iterative methods by solving exactly four initial value problems for Eq. (7). The rationale for this approach can be found in (Omel’chenko 2023, Section 4) and is repeated for completeness in Remark 3 in Appendix. Below we describe its concrete implementation in the case of formula (10).

We assume that the spatial domain $[0,2\pi ]$ is discretised with *N* points, $x_j, j=1,2,\ldots N$ and that the functions *U*(*x*, *t*) and *W*(*x*, *t*) are replaced with their grid counterparts $u_j(t) = U(x_j,t)$ and $w_j(t) = W(x_j,t)$, respectively. Then, given a set of functions $w_j(t)$, we calculate the corresponding functions $u_j(t)$ by performing the following four steps.

(i) We (somewhat arbitrarily) choose three initial conditions $u_j^1(0)=-0.95$, $u_j^2(0)=0$, $u_j^3(0)=0.95$ and solve Eq. (7) with these initial conditions to obtain solutions $u_j^k(t)$, $j=1,\ldots N$; $k=1,2,3$. In Omel’chenko (2023) (see also Proposition 2 in the Appendix) it is shown that for $\gamma > 0$ these solutions lie in the open unit disc ${\mathbb {D}}$.

(ii) Since at each point in space the Poincaré map of Eq. (7) with $2\pi$-periodic coefficients coincides with a Möbius map ${\mathcal {M}}(u)$ (see Omel’chenko 2023 for detail), we can use the relations $u_j^k(2\pi ) = {\mathcal {M}}_j( u_j^k(0) )$, $k=1,2,3$, to reconstruct these maps ${\mathcal {M}}_j$, $j=1,\ldots N$. The corresponding formulas are given in Omel’chenko (2023, Section 4).

(iii) Now that the Möbius maps ${\mathcal {M}}_j(u)$ are known, their fixed points can be found by solving $u_j^*= {\mathcal {M}}_j(u_j^*)$ for each *j*. This equation is equivalent to a complex quadratic equation, therefore in general it has two solutions in the complex plane. For $\gamma > 0$, only one of these solutions lies in the unit disc ${\mathbb {D}}$.

(iv) Using the fixed points $u_j^*\in {\mathbb {D}}$ of the Möbius maps ${\mathcal {M}}_j(u)$ as an initial condition in Eq. (7) (i.e. setting $u(x_j,0)=u_j^*$) and integrating (7) for a fourth time to $t=2\pi$ we obtain the grid counterpart of a $2\pi$-periodic solution, *U*(*x*, *t*), that lies entirely in the unit disc ${\mathbb {D}}$.

We now use this result to show how to derive a self-consistency equation, the solution of which allows us to determine a $2\pi$-periodic solution of Eq. (6).

### Self-consistency equation

Supposing that Eq. (6) has a $2\pi$-periodic solution, then using formula (8) we can calculate the corresponding function *W*(*x*, *t*). On the other hand, using formula (10) we can recover *u*(*x*, *t*). Then the new and the old expressions of *u*(*x*, *t*) will agree with each other if and only if the function *W*(*x*, *t*) satisfies a self-consistency equation

11 $$\begin{aligned} W(x,t) = \frac{\displaystyle \kappa }{\displaystyle 2\omega } {\mathcal {K}} H_n\left( {\mathcal {U}}\left( W(x,t), \frac{\displaystyle \eta _0 + i \gamma }{\displaystyle 2\omega }, \omega \right) \right) , \end{aligned}$$

obtained by inserting (10) into (8). In the following we consider Eq. (11) as a separate equation which must be solved with respect to *W*(*x*, *t*) and $\omega$. Note that the unknown field *W*(*x*, *t*) has a problem-specific meaning: It is proportional to the current entering a neuron at position *x* at time *t* due to the activity of all other neurons in the network. The use of self-consistency arguments to study infinite networks of oscillators goes back to Kuramoto (1984), Strogatz (2000) and Shima and Kuramoto (2004), but such approaches have always focused on steady states, whereas we consider periodic solutions here.

Note that from a computational point of view, the self-consistency Eq. (11) has many advantages. It allows us to reduce the dimensionality of the problem at least in the case of special coupling kernels with finite number of Fourier harmonics (see Sect. 2.3). Moreover, its structure is convenient for parallelization, since the computations of operator ${\mathcal {U}}$ at different points *x* are performed independently. Finally, the main efficiency is due to the fact that the computation of ${\mathcal {U}}$ is performed in non-iterative way.

In the next proposition, we will prove some properties of the solutions of Eq. (11), which will be used later in Sect. 2.5.

#### Proposition 1

Let the pair $( W(x,t), \omega )$ be a solution of the self-consistency Eq. (11) and let *U*(*x*, *t*) be defined by (10). Then

$$\begin{aligned} \left| \exp \left( \int _0^{2\pi } M(x,t) \textrm{d}t \right) \right| < 1 \end{aligned}$$

where

12 $$\begin{aligned} M(x,t)= & {} \frac{\displaystyle i}{\displaystyle \omega } \left[ ( \kappa {\mathcal {K}} H_n(U) + \eta _0 + i \gamma ) ( 1 + U(x,t) )\right. \nonumber \\{} & {} + \left. 1 - U(x,t) \right] . \end{aligned}$$

#### Proof

For every fixed $x\in [0,2\pi ]$, the function *U*(*x*, *t*) yields a stable $2\pi$-periodic solution of the complex Riccati Eq. (7). The linearization of Eq. (7) around this solution reads

$$\begin{aligned} \frac{\displaystyle \textrm{d}v}{\displaystyle \textrm{d}t} = M(x,t) v, \end{aligned}$$

where

$$\begin{aligned} M(x,t)= & {} 2 i \left( W(x,t) + \zeta + \frac{\displaystyle 1}{\displaystyle 2\omega } \right) \nonumber \\{} & {} + 2 i \left( W(x,t) + \zeta - \frac{\displaystyle 1}{\displaystyle 2\omega } \right) U(x,t). \end{aligned}$$

Moreover, using (8) and (9), we can show that the above expression determines a function identical to the function *M*(*x*, *t*) in (12). Recalling that *U*(*x*, *t*) is not only a stable but also an asymptotically stable solution of Eq. (7), see Remark 1 in Appendix, we conclude that the corresponding Floquet multiplier lies in the open unit disc ${\mathbb {D}}$. This ends the proof.  $\square$

### Numerical implementation

Equation (11) describes a periodic orbit, and since Eq. (7) is autonomous we need to append a pinning condition in order to select a specific solution of Eq. (11). For a solution of the type shown in Fig. 1 we choose

13 $$\begin{aligned} \int _0^{2\pi } \textrm{d}x \int _0^{2\pi } W(x,t) \sin (2 t) \textrm{d}t = 0. \end{aligned}$$

In the following we focus on the case of the cosine coupling (3). It is straight-forward to show that $({\mathcal {K}} H_n)(x)$ can be written as a linear combination of $1,\cos {x}$ and $\sin {x}$. However, the system is translationally invariant in *x*, and we can eliminate this invariance from Eq. (11) by restricting this equation to its invariant subspace $\textrm{Span}\{ 1, \sin x \}$. Then, taking into account that the function *W*(*x*, *t*) is real, we seek an approximate solution of the system (11), (13) using a Fourier-Galerkin ansatz

14 $$\begin{aligned} W(x,t) = \sum \limits _{m=0}^{2F} ( v_m + w_m \sin x ) \psi _m(t) \end{aligned}$$

where $v_m$ and $w_m$ are real coefficients and $\psi _m(t)$ are trigonometric basis functions

$$\begin{aligned} \psi _0(t)= & {} 1,\\ \psi _m(t)= & {} \sqrt{2} \cos (n t),\quad \text{ if }\quad m = 2 n\quad \text{ with }\quad n\in {\mathbb {N}},\\ \psi _m(t)= & {} \sqrt{2} \sin (n t),\quad \text{ if }\quad m = 2 n -1\quad \text{ with }\quad n\in {\mathbb {N}}. \end{aligned}$$

Our typical choice of the number of harmonics in (14) is $F = 10$. To exactly represent *W*(*x*, *t*) in (14) would require an infinite number of terms in the series, so using a finite value of *F* introduces an approximation in our calculations. However, the excellent agreement between our calculations with $F=10$ and those from full simulations of (4) (shown below) indicate that such an approximation is justified.

Using the scalar product

$$\begin{aligned} \langle u, v \rangle = \frac{\displaystyle 1}{\displaystyle (2\pi )^2} \int _0^{2\pi } \textrm{d}x \int _0^{2\pi } u(x,t) v(x,t) \textrm{d}t \end{aligned}$$

we project Eq. (11) on different spatio-temporal Fourier modes to obtain the system

15 $$\begin{aligned} v_m= & {} \frac{\displaystyle \kappa }{\displaystyle 2\omega } \left\langle H_n\left( {\mathcal {U}}\left( W(x,t), \frac{\displaystyle \eta _0 + i \gamma }{\displaystyle 2\omega }, \omega \right) \right) , \psi _m(t) \right\rangle , \nonumber \\ \end{aligned}$$

16 $$\begin{aligned} w_m= & {} \frac{\displaystyle \kappa A}{\displaystyle 2\omega } \left\langle H_n\left( {\mathcal {U}}\left( W(x,t), \frac{\displaystyle \eta _0 + i \gamma }{\displaystyle 2\omega }, \omega \right) \right) , \right. \nonumber \\{} & {} \left. \psi _m(t) \sin x \right\rangle , \end{aligned}$$

for $m=0,1,\ldots , 2F$. Equations (15) and (16), together with (13), are a set of $2(2F+1)+1=4F+3$ equations for the $4F+3$ unknowns $v_0,v_1,\ldots , v_{2F},w_0,w_1,\ldots , w_{2F},\omega$, which must be solved simultaneously. We solve them using Newton’s method and find convergence within 3 or 4 iterations.

In simple terms, suppose we have somewhat accurate estimates of $v_0,v_1,\ldots , v_{2F},w_0,w_1,\ldots , w_{2F},\omega$. These can be inserted into (14) to calculate the function *W*(*x*, *t*). Then one can calculate a periodic solution of Eq. (7) with the specified *W*(*x*, *t*) by formula (10) and finally calculate $H_n( {\mathcal {U}})$ and insert this into (15) and (16) and perform the projections. We want the difference between the new values of $v_0,v_1,\ldots , v_{2F},w_0,w_1,\ldots , w_{2F}$ and the initial values of them to be zero, and for (13) to hold. This determines the $4F+3$ equations we need to solve. The solutions of these equations can be followed as a parameter is varied in the standard way (Laing 2014b). Note that these calculations involve discretising the spatial domain with *N* points. However, the number of unknowns ($4F+3$) is significantly less than *N*.

### Results

**Fig. 2:**
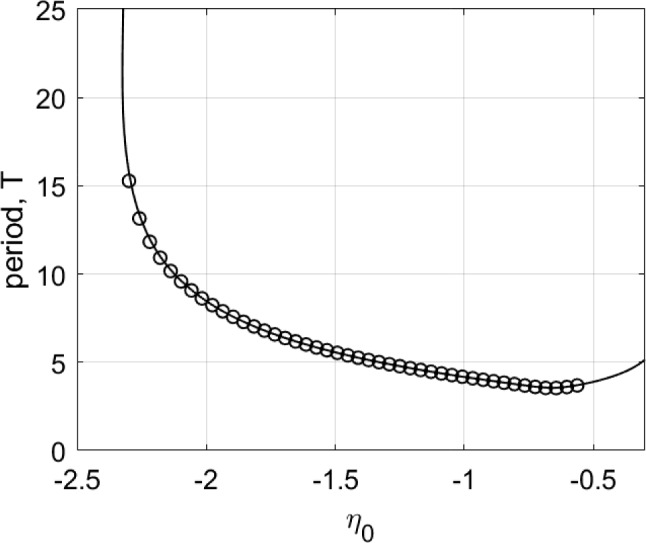
Period of the type of solution shown in Fig. 1 as a function of $\eta _0$ (solid curve). The circles show values measured from direct simulations of Eq. (4). Other parameters: $A=-5$, $\kappa =1$, $\gamma =0.01$

The results of following the solution shown in Fig. 1 as $\eta _0$ is varied are shown in Fig. 2, along with values measured from direct simulations of Eq. (4). The period seems to become arbitrarily large as $\eta _0$ approaches $-2.32$, and the solution approaches a heteroclinic connection, spending more and more time near two symmetric states which are mapped to one another under the transformation $x\rightarrow -x$. The solution becomes unstable at $\eta _0\approx -0.5$ through a subcritical torus (or secondary Hopf) bifurcation. (This was determined by finding the Floquet multipliers of the periodic solution in the conventional way; results not shown.) For these calculations we used $N=256$ spatial points.

**Fig. 3:**
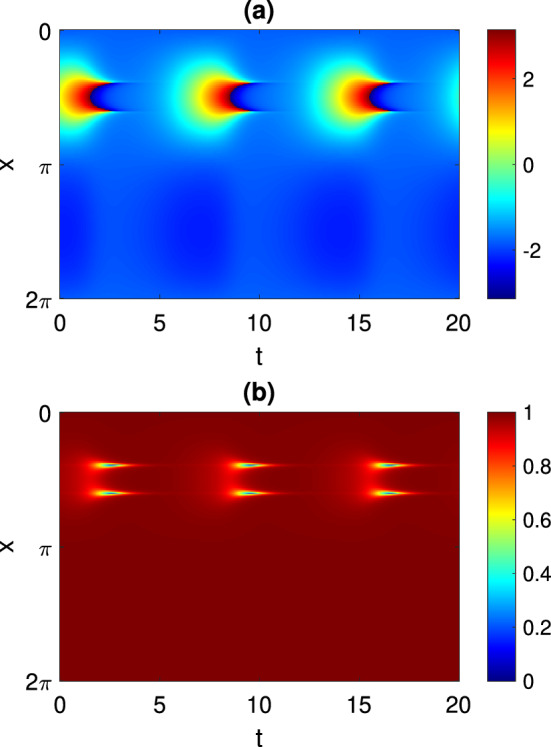
Another periodic solution of Eq. (4). **a** $\arg {(z(x,t))}$. **b** |*z*(*x*, *t*)|. Parameters: $A=-5$, $\eta _0=-2.5$, $\kappa =1$, $\gamma =0.01$

**Fig. 4:**
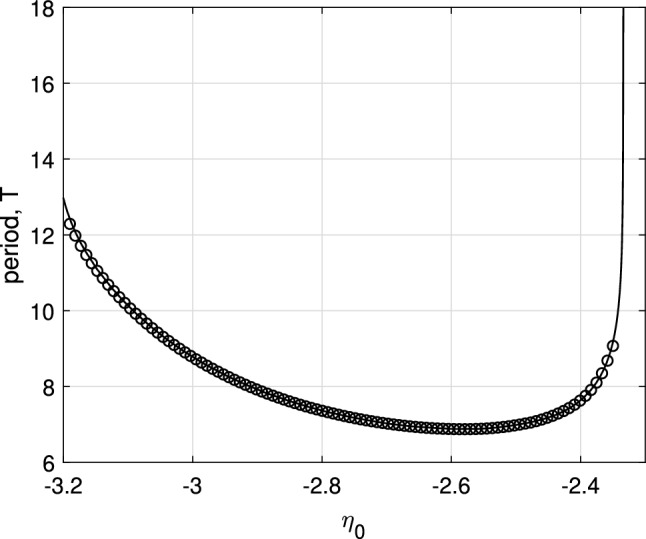
Period of the type of solution shown in Fig. 3 as a function of $\eta _0$ (solid curve). The circles show values measured from direct simulations of Eq. (4). Other parameters: $A=-5$, $\kappa =1$, $\gamma =0.01$

For more negative values of $\eta _0$ than those shown in Fig. 2, another type of periodic solution is stable: see Fig. 3. Such a solution does not have the spatio-temporal symmetry of the solution shown in Fig. 1. However, we can follow it in just the same way as the parameter $\eta _0$ is varied, and we obtain the results shown in Fig. 4. This periodic orbit appears to be destroyed in a supercritical Hopf bifurcation as $\eta _0$ is decreased through approximately $-3.2$, and become unstable to a wandering pattern at $\eta _0$ is increased through approximately $-2.34$.

Note that the left asymptote in Fig. 2 coincides with the right asymptote in Fig. 4. On the other hand, we note that two patterns shown in Figs. 1 and 3 have different spatiotemporal symmetries, therefore due to topological reasons they cannot continuously transform into each other. Similar bifurcation diagrams where parameter ranges of two patterns with different symmetries are separated by heteroclinic or homoclinic bifurcations were found for non-locally coupled Kuramoto-type phase oscillators (Omel’chenko 2020) and seem to be a general mechanism which, however, needs additional investigation.

### Stability of breathing bumps

Given a *T*-periodic solution *a*(*x*, *t*) of Eq. (4), we can perform its linear stability analysis, using the approach proposed in Omel’chenko (2022). Before doing this, we write

$$\begin{aligned} H_n(z)=a_nC_0+2\text{ Re }[D_n(z)] \end{aligned}$$

where

$$\begin{aligned} D_n(z)=a_n\sum _{q=1}^n C_qz^q, \end{aligned}$$

to emphasise that $H_n(z)$ is always real. Now, we insert the ansatz $z(x,t) = a(x,t) + v(x,t)$ into Eq. (4) and linearize the resulting equation with respect to small perturbations *v*(*x*, *t*). Thus, we obtain a linear integro-differential equation

17 $$\begin{aligned} \frac{\displaystyle \partial v}{\displaystyle \partial t} = \mu (x,t) v + \kappa \frac{\displaystyle i ( 1 + a(x,t) )^2 }{\displaystyle 2} {\mathcal {K}} \left( D_n'(a) v + \overline{D_n'(a)} {\overline{v}} \right) , \nonumber \\ \end{aligned}$$

where

18 $$\begin{aligned} \mu (x,t)= & {} [ i (\eta _0 + \kappa {\mathcal {K}} H_n(a)) - \gamma ] ( 1 + a(x,t) ) \nonumber \\{} & {} + i ( 1- a(x,t) ) \end{aligned}$$

and

$$\begin{aligned} D_n'(z) = \frac{\displaystyle \textrm{d}}{\displaystyle \textrm{d}z} D_n(z) = a_n \sum \limits _{q=1}^n q C_q z^{q-1}. \end{aligned}$$

Note that Eq. (17) coincides with Eq. (5.1) from Omel’chenko and Laing (2022), except that the coefficients *a*(*x*, *t*) and $\mu (x,t)$ are now time-dependent. Since Eq. (17) contains the complex-conjugated term ${\overline{v}}$, it is convenient to consider this equation along with its complex-conjugate

$$\begin{aligned} \frac{\displaystyle \partial {\overline{v}}}{\displaystyle \partial t} = {\overline{\mu }}(x,t) {\overline{v}} - \kappa \frac{\displaystyle i ( 1 + {\overline{a}}(x,t) )^2 }{\displaystyle 2} {\mathcal {K}} \left( D_n'(a) v + \overline{D_n'(a)} {\overline{v}} \right) . \end{aligned}$$

This pair of equations can be written in the operator form

19 $$\begin{aligned} \frac{\displaystyle \textrm{d}V}{\displaystyle \textrm{d}t} = {\mathcal {A}}(t) V + {\mathcal {B}}(t) V, \end{aligned}$$

where $V(t) = ( v_1(t), v_2(t) )^\textrm{T}$ is a function with values in $C_{\textrm{per}}([0,2\pi ]; {\mathbb {C}}^2)$, and

$$\begin{aligned} {\mathcal {A}}(t) V = \left( \begin{array}{cc} \mu (x,t) &{} \quad 0 \\ 0 &{} \quad {\overline{\mu }}(x,t) \end{array} \right) \left( \begin{array}{c} v_1 \\ v_2 \end{array} \right) , \end{aligned}$$

and

20 $$\begin{aligned} {\mathcal {B}}(t) V= & {} \frac{\displaystyle i \kappa }{\displaystyle 2} \left( \begin{array}{cc} ( 1 + a(x,t) )^2 &{} \quad 0 \\ 0 &{} \quad - ( 1 + {\overline{a}}(x,t) )^2 \end{array} \right) \nonumber \\{} & {} \times \left( \begin{array}{c} {\mathcal {K}} \left( D_n'(a) v_1 \right) \\ {\mathcal {K}} \left( \overline{D_n'(a)} v_2 \right) \end{array} \right) . \end{aligned}$$

For every fixed *t* the operators ${\mathcal {A}}(t)$ and ${\mathcal {B}}(t)$ are linear operators from $C_{\textrm{per}}([0,2\pi ]; {\mathbb {C}}^2)$ into itself. Moreover, they both depend continuously on *t* and thus their norms are uniformly bounded for all $t\in [0,T]$.

Recall that the question of linear stability of *a*(*x*, *t*) in Eq. (4) is equivalent to the question of linear stability of the zero solution in Eq. (17), and hence to the question of linear stability of the zero solution in Eq. (19). Moreover, using the general theory of periodic differential equations in Banach spaces, see Daleckiĭ and Kreĭn (2002, Chapter V), the last question can be reduced to the analysis of the spectrum of the monodromy operator ${\mathcal {E}}(T)$ defined by the operator exponent

$$\begin{aligned} {\mathcal {E}}(t) = \exp \left( \int _0^t ( {\mathcal {A}}(t') + {\mathcal {B}}(t') ) dt' \right) . \end{aligned}$$

The analysis of Eq. (19) in the case when ${\mathcal {A}}(t)$ is a matrix multiplication operator and ${\mathcal {B}}(t)$ is an integral operator similar to (20) has been performed in (Omel’chenko 2022, Section 4). Repeating the same arguments we can demonstrate that the spectrum of the monodromy operator ${\mathcal {E}}(T)$ is bounded and symmetric with respect to the real axis of the complex plane. Moreover, it consists of two qualitatively different parts:

(i) the essential spectrum, which is given by the formula

21 $$\begin{aligned} \sigma _\textrm{ess} = \left\{ \exp \left( \int _0^T \mu (x,t) dt \right) : x\in [0,2\pi ] \right\} \cup \{\mathrm {c.c.}\} \nonumber \\ \end{aligned}$$

(ii) the discrete spectrum $\sigma _\textrm{disc}$ that consists of finitely many isolated eigenvalues $\lambda$, which can be found using a characteristic integral equation, as explained in ( Omel’chenko 2022, Section 4).

Note that if *a*(*x*, *t*) is obtained by solving the self-consistency Eq. (11) and hence it satisfies

$$\begin{aligned} a(x,t/\omega ) = U(x,t) = {\mathcal {U}}\left( W(x,t), \frac{\displaystyle \eta _0 + i \gamma }{\displaystyle 2\omega }, \omega \right) , \end{aligned}$$

where $(W(x,t),\omega )$ is a solution of Eq. (11), then we can use Proposition 1 and formula (18) to show

$$\begin{aligned} \left| \exp \left( \int _0^T \mu (x,t) dt \right) \right| < 1\quad \text{ for } \text{ all }\quad x\in [0,2\pi ]. \end{aligned}$$

In this case, the essential spectrum $\sigma _\textrm{ess}$ lies in the open unit disc ${\mathbb {D}}$ and therefore it cannot contribute to any linear instability of the zero solution of Eq. (19).

**Fig. 5:**
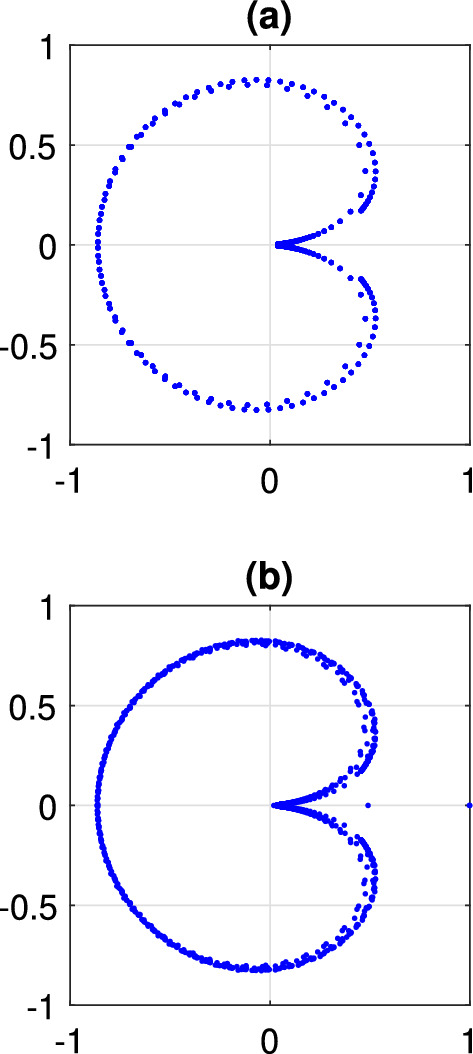
**a** The essential spectrum given by (21) for the periodic solution shown in Fig. 1. **b** Floquet multipliers of the same periodic solution found using the technique explained at the start of Sect. 2. For both calculations the spatial domain has been discretized using 512 evenly spaced points

To illustrate the usefulness of formula (21), in Fig. 5a we plot the essential spectrum for the periodic solution shown in Fig. 1. In Fig. 5b we show the Floquet multipliers of the same periodic solution, where we have found the solution and its stability in the conventional way, of discretizing the domain and finding a periodic solution of a large set of coupled ordinary differential equations. In panel (b) we see several real Floquet multipliers that do not appear in panel (a); these are presumably part of the discrete spectrum. Note that calculating the discrete spectrum by the method of Omel’chenko (2022, Section 4) is numerically difficult, so we do not do that here.

### Formula for firing rates

One quantity of interest in a network of model neurons such as (1) is their firing rate. The firing rate of the *k*th neuron is defined by

$$\begin{aligned} f_k = \frac{\displaystyle 1}{\displaystyle 2\pi } \left\langle \frac{\displaystyle \textrm{d}\theta _k}{\displaystyle \textrm{d}t} \right\rangle _\textrm{T}, \end{aligned}$$

where the angled brackets $\langle \cdot \rangle _\textrm{T}$ indicate a long-time average. In the case of large *N*, we can also consider the average firing rate

22 $$\begin{aligned} f(x) = \frac{\displaystyle 1}{\displaystyle \#\{ k: | x_k - x |< \pi /\sqrt{N} \}} \sum \limits _{| x_k - x | < \pi /\sqrt{N}} f_k, \end{aligned}$$

where $x_k = 2\pi k/N$ is the spatial positions of the *k*th neuron and the averaging takes place over all neurons in the $(\pi /\sqrt{N})$-vicinity of the point $x\in [0,2\pi ]$. Note that while the individual firing rates $f_k$ are usually randomly distributed due to the randomness of the excitability parameters $\eta _k$, the average firing rate *f*(*x*) converges to a continuous (and even smooth) function for $N\rightarrow \infty$. Moreover, the exact prediction of the limit function *f*(*x*) can be given, using only the corresponding solution *z*(*x*, *t*) of Eq. (4). To show this, we write Eq. (1) as

23 $$\begin{aligned} \frac{\displaystyle \textrm{d}\theta _k}{\displaystyle \textrm{d}t} = \textrm{Re}\left\{ 1 - e^{i \theta _k} + ( \eta _k + \kappa I_k ) ( 1 + e^{i \theta _k} ) \right\} . \end{aligned}$$

We recall that in deriving (4) from (1) we introduce a probability distribution $\rho (\theta ,x,\eta ,t)$ which satisfies a continuity equation (Laing 2015; Omel’chenko et al. 2014; Laing 2014a). At a given time *t*, $\rho (\theta ,x,\eta ,t)\textrm{d}\theta \textrm{d}\eta \textrm{d}x$ is the probability that a neuron with a position in $[x, x + dx]$ and intrinsic drive in $[\eta , \eta + \textrm{d}\eta ]$ has its phase in $[\theta , \theta + d\theta ]$. Moreover, in the case of the Lorentzian distribution of parameters $\eta _k$, the probability distribution $\rho (\theta ,x,\eta ,t)$ satisfies the relations

24 $$\begin{aligned}{} & {} \int _{-\infty }^\infty \textrm{d} \eta \int _0^{2\pi } \rho (\theta ,x,\eta ,t) e^{i \theta } \textrm{d}\theta = z(x,t), \end{aligned}$$

25 $$\begin{aligned}{} & {} \int _{-\infty }^\infty \textrm{d}\eta \int _0^{2\pi } \eta \rho (\theta ,x,\eta ,t) e^{i \theta } \textrm{d}\theta = (\eta _0 + i \gamma ) z(x,t), \nonumber \\ \end{aligned}$$

which are obtained by a standard contour integration in the complex plane (Ott and Antonsen 2008). The relation (24) has been already used to calculate the continuum limit analog of (2)

$$\begin{aligned} I(x,t) = \int _0^{2\pi } K(x-y) H_n(z(y,t)) \textrm{d}y = {\mathcal {K}} H_n(z). \end{aligned}$$

Inserting this instead of $I_k(t)$ into Eq. (23) and replacing the time and index averaging in (22) with the corresponding averaging over the probability density, we obtain

$$\begin{aligned} f(x)= & {} \lim _{\tau \rightarrow \infty }\frac{1}{2\pi \tau }\int _0^\tau \int _{-\infty }^\infty \int _0^{2\pi } \\ {}{} & {} \textrm{Re} \left\{ 1 - e^{i \theta } + ( \eta + \kappa I(x,t) ) ( 1 + e^{i \theta } ) \right\} \\ {}{} & {} \rho (\theta ,x,\eta ,t) \textrm{d}\theta \ \textrm{d}\eta \ \textrm{d}t. \end{aligned}$$

The two inner integrals in the above formula can be simplified using the relations (24), (25) and the standard normalization condition for $\rho (\theta ,x,\eta ,t)$. Thus we obtain

$$\begin{aligned} f(x)= & {} \lim _{\tau \rightarrow \infty }\frac{1}{2\pi \tau }\int _0^\tau \textrm{Re} \left\{ 1 - z(x,t) \right. \\ {}{} & {} \left. + ( \eta _0 + i \gamma + \kappa I(x,t) ) ( 1 + z(x,t) ) \right\} \textrm{d}t. \end{aligned}$$

Moreover, if $z = a(x,t)$ is a *T*-periodic solution of Eq. (4), then the long-time average is the same as an average over one period. So, in the periodic case, the continuum limit average firing rate equals

26 $$\begin{aligned} f(x)= & {} \frac{\displaystyle 1}{\displaystyle 2\pi T} \int _0^T \textrm{Re} \left\{ 1 - a(x,t) \right. \nonumber \\{} & {} \left. + ( \eta _0 + i \gamma + \kappa I(x,t) ) ( 1 + a(x,t) ) \right\} \textrm{d}t. \end{aligned}$$

(Note that with simple time rescaling formula (26) can be rewritten in terms of a $2\pi$-periodic solution of the complex Riccati Eq. (7), $u(x,t) = a(x,t/\omega )$.) The expression (26) is different from the firing rate expression given in Sect. 1, but both are equally valid.

**Fig. 6:**
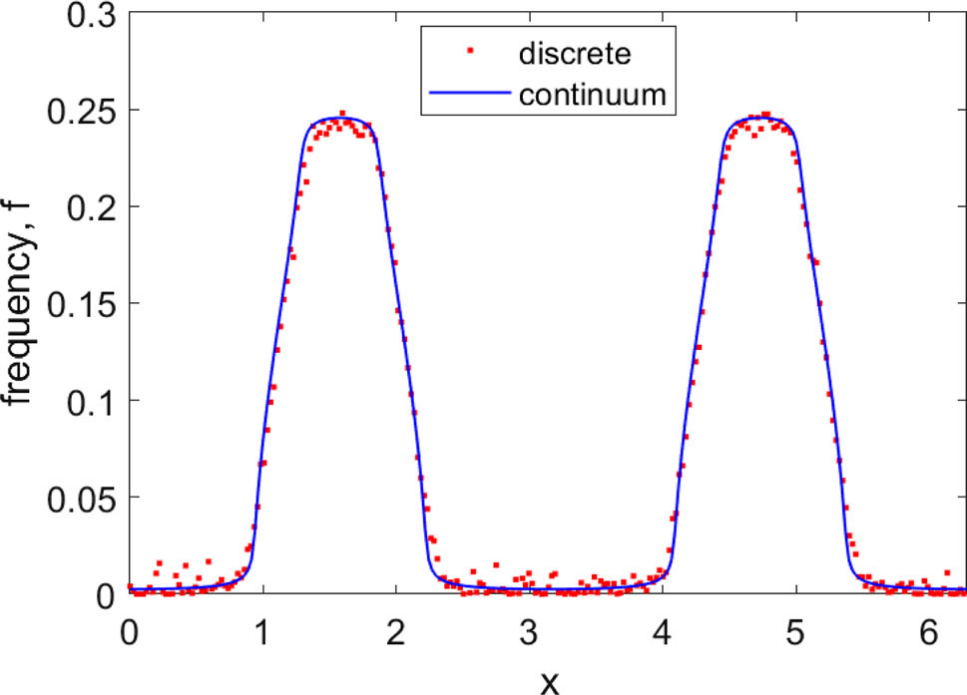
Average firing rate for a pattern like that shown in Fig. 1. The curve shows *f*(*x*) as given by (26). The dots show values measured from direct simulations of Eq. (1). For the discrete simulation, $N=2^{14}$ neurons were used and the average frequency profile, $\{f_j\}, j=1,2\dots N$, was convolved with a spatial Gaussian filter of standard deviation 0.01 before plotting. For clarity, not all points are shown. Other parameters: $A=-5$, $\eta _0=-0.9$, $\kappa =1$, $\gamma =0.01$

Results for a pattern like that shown in Fig. 1 are given in Fig. 6, where we show both *f*(*x*) (from (26)) and values extracted from a long simulation of Eq. (1). The agreement is very good.

## Other models

We now demonstrate how the approach presented above can be applied to various other neural models.

### Delays

Delays in neural systems are ubiquitous due to the finite velocity at which action potentials propagate as well as to both dendritic and synaptic processing (Roxin et al. 2005; Coombes and Laing 2009; Atay and Hutt 2006; Devalle et al. 2018). Here we assume that all $I_j(t)$ are delayed by a fixed amount $\tau$, i.e. we have Eq. (1) but we replace (2) by

27 $$\begin{aligned} I_j(t) = \frac{\displaystyle 2\pi }{\displaystyle N} \sum \limits _{k=1}^N K_{jk} P_n(\theta _k(t-\tau )). \end{aligned}$$

The mean field equation is now

28 $$\begin{aligned} \frac{\displaystyle \partial z}{\displaystyle \partial t}= & {} \frac{\displaystyle (i \eta _0 - \gamma ) ( 1 + z )^2 - i ( 1- z )^2 }{\displaystyle 2} \nonumber \\{} & {} + \kappa \frac{\displaystyle i ( 1 + z )^2 }{\displaystyle 2} {\mathcal {K}} H_n(z(x,t-\tau )). \end{aligned}$$

We can write this equation in the same form as Eq. (7) but now

29 $$\begin{aligned} W(x,t) = \frac{\displaystyle \kappa }{\displaystyle 2\omega } {\mathcal {K}} H_n(u(x,t-\omega \tau )), \end{aligned}$$

and the corresponding self-consistency equation is also time-delayed

$$\begin{aligned} W(x,t) = \frac{\displaystyle \kappa }{\displaystyle 2\omega } {\mathcal {K}} H_n\left( {\mathcal {U}}\left( W(x,t - \omega \tau ), \frac{\displaystyle \eta _0 + i \gamma }{\displaystyle 2\omega }, \omega \right) \right) . \end{aligned}$$

We expand *W*(*x*, *t*) as in (14), and given *W*(*x*, *t*), we find the relevant $2\pi$-periodic solution of Eq. (7) as above. The only difference comes in evaluating the projections (15) and (16). Instead of using $H_n(U(x,t))$ in the scalar products, we need to use $H_n(U(x,t-\omega \tau ))$.

Since *U*(*x*, *t*) is $2\pi$-periodic in time, we can evaluate it at any time using just its values for $t\in [0,2\pi ]$. Specifically,

30 $$\begin{aligned} U(x,t-\omega \tau ) = {\left\{ \begin{array}{ll} U(x,2\pi +t-\omega \tau ), &{}\quad 0\le t\le \omega \tau , \\ U(x,t-\omega \tau ), &{} \quad \omega \tau < t\le 2\pi . \end{array}\right. }\nonumber \\ \end{aligned}$$

Note that this approach would also be applicable if one had a distribution of delays (Lee et al. 2009; Laing and Longtin 2003) or even state-dependent delays (Keane et al. 2019).

**Fig. 7:**
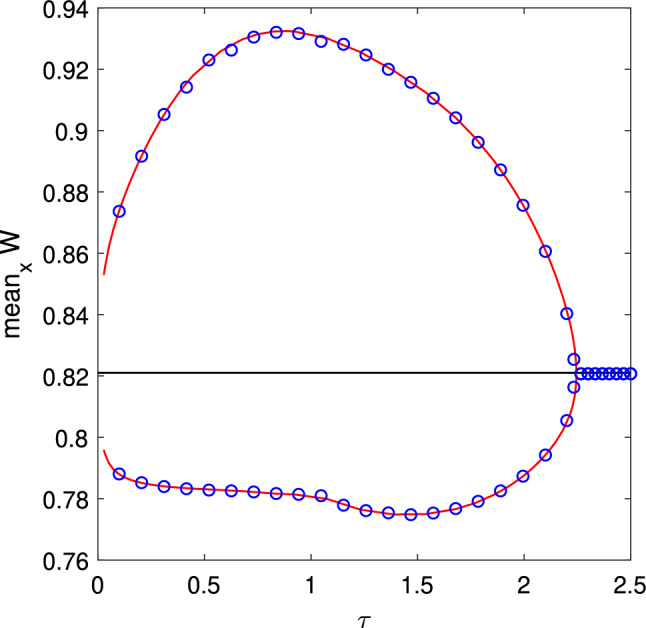
The vertical axis relates to averaging *W*(*x*, *t*) over *x*. For a periodic solution, the maximum and minimum over one period of this quantity is plotted. The black horizontal line corresponds to the steady state which is stable at $\tau =2.5$. Circles: measured from direct simulations of Eq. (28). Other parameters: $A=-5$, $\eta _0=-2$, $\kappa =1$, $\gamma =0.1$

As an example, we show in Fig. 7 the results of varying the delay $\tau$ on a solution of the form shown in Fig. 3. Increasing $\tau$ leads to the destruction of the periodic solution in an apparent supercritical Hopf bifurcation.

### Two populations

Neurons fall into two major categories: excitatory and inhibitory. A model consisting of a single population with a coupling function of the form (3) is often used as an approximation to a two-population model (Esnaola-Acebes et al. 2017). Here we consider a two population model which supports a travelling wave. The mean field equations are

31 $$\begin{aligned} \frac{\displaystyle \partial u}{\displaystyle \partial t}= & {} \frac{\displaystyle (i \eta _u - \gamma ) ( 1 + u )^2 - i ( 1- u )^2 }{\displaystyle 2} \nonumber \\{} & {} + \frac{\displaystyle i ( 1 + u )^2 }{\displaystyle 2} \left[ w_{\textrm{ee}}{\mathcal {K}} H_n(u)-w_{\textrm{ei}}{\mathcal {K}} H_n(v)\right] , \end{aligned}$$

32 $$\begin{aligned} \frac{\displaystyle \partial v}{\displaystyle \partial t}= & {} \frac{\displaystyle (i \eta _v - \gamma ) ( 1 + v )^2 - i ( 1- v )^2 }{\displaystyle 2} \nonumber \\{} & {} + \frac{\displaystyle i ( 1 + v )^2 }{\displaystyle 2} \left[ w_{\textrm{ie}}{\mathcal {K}} H_n(u)-w_{\textrm{ii}}{\mathcal {K}} H_n(v)\right] \end{aligned}$$

where *u*(*x*, *t*) is the complex-valued order parameter for the excitatory population and *v*(*x*, *t*) is that for the inhibitory population. The non-negative connectivity kernel between and within populations is the same:

$$\begin{aligned} K(x)=\frac{1}{2\pi }(1+\cos {x}) \end{aligned}$$

and there are four connection strengths within and between populations: $w_{\textrm{ee}}$, $w_{\textrm{ei}}$, $w_{\textrm{ie}}$ and $w_{\textrm{ii}}$. Similar models have been studied in Blomquist et al. (2005), Pinto and Ermentrout (2001).

For some parameter values, such a system supports a travelling wave with a constant profile. Such a wave can be found very efficiently using the techniques discussed here, and that was done for a travelling chimera in Omel’chenko (2023). However, here we consider a slightly different case: that where the mean drive to the excitatory population, $\eta _u$, is spatially modulated. We thus write

$$\begin{aligned} \eta _u = \eta _0 + \epsilon \sin {x}. \end{aligned}$$

**Fig. 8:**
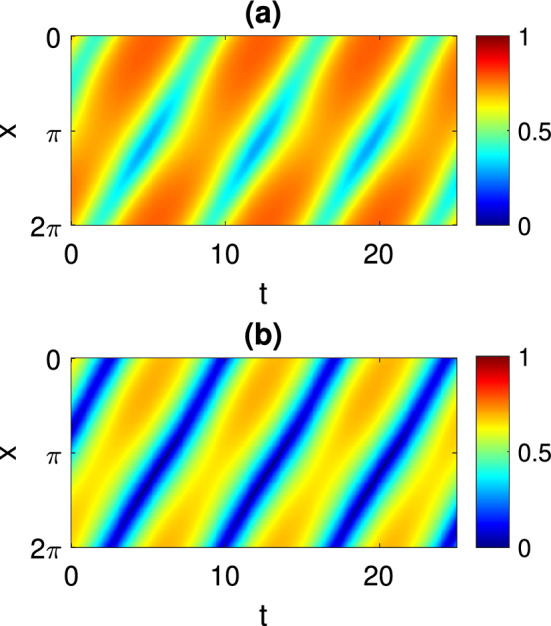
A modulated travelling wave solution of Eqs. (31)–(32). **a** |*u*|; **b** |*v*|. Other parameters: $\eta _0=0.1$, $\eta _v=0.1$, $\epsilon =0.01$, $\gamma =0.03$, $w_{\textrm{ee}}=1$, $w_{\textrm{ei}}=0.7$, $w_{\textrm{ie}}=0.3$, $w_{\textrm{ii}}=0.1$

For small $|\epsilon |$ the travelling wave persists, but not with a constant profile. An example is shown in Fig. 8. Note that such a solution is periodic in time.

By rescaling time we can write Eqs. (31)–(32) as

33 $$\begin{aligned} \frac{\displaystyle \partial {\tilde{u}}}{\displaystyle \partial t}= & {} i \left( w_{\textrm{ee}}W_u-w_{\textrm{ei}}W_v + \zeta _u - \frac{\displaystyle 1}{\displaystyle 2\omega } \right) \nonumber \\{} & {} + 2 i \left( w_{\textrm{ee}}W_u-w_{\textrm{ei}}W_v + \zeta _u + \frac{\displaystyle 1}{\displaystyle 2\omega } \right) {\tilde{u}} \nonumber \\{} & {} + i \left( w_{\textrm{ee}}W_u-w_{\textrm{ei}}W_v + \zeta _u - \frac{\displaystyle 1}{\displaystyle 2\omega } \right) {\tilde{u}}^2, \end{aligned}$$

34 $$\begin{aligned} \frac{\displaystyle \partial {\tilde{v}}}{\displaystyle \partial t}= & {} i \left( w_{\textrm{ie}}W_u-w_{\textrm{ii}}W_v + \zeta _v - \frac{\displaystyle 1}{\displaystyle 2\omega } \right) \nonumber \\{} & {} + 2 i \left( w_{\textrm{ie}}W_u-w_{\textrm{ii}}W_v + \zeta _v + \frac{\displaystyle 1}{\displaystyle 2\omega } \right) {\tilde{v}} \nonumber \\{} & {} + i \left( w_{\textrm{ie}}W_u-w_{\textrm{ii}}W_v + \zeta _v - \frac{\displaystyle 1}{\displaystyle 2\omega } \right) {\tilde{v}}^2, \end{aligned}$$

where $${\tilde{u}}(x,t)\equiv u(x,t/\omega )$$, $${\tilde{v}}(x,t)\equiv v(x,t/\omega )$$,

35 $$\begin{aligned} W_u(x,t)= & {} \frac{\displaystyle 1}{\displaystyle 2\omega } {\mathcal {K}} H_n(u), \end{aligned}$$

36 $$\begin{aligned} W_v(x,t)= & {} \frac{\displaystyle 1}{\displaystyle 2\omega } {\mathcal {K}} H_n(v) \end{aligned}$$

and

$$\begin{aligned} \zeta _u = \frac{\displaystyle \eta _u + i \gamma }{\displaystyle 2\omega } = \frac{\displaystyle \eta _0 + \epsilon \sin {x} + i \gamma }{\displaystyle 2\omega }, \qquad \zeta _v = \frac{\displaystyle \eta _v + i \gamma }{\displaystyle 2\omega }. \end{aligned}$$

In the same way as above, we can derive self-consistency equations of the form (11) for $W_u(x,t)$ and $W_v(x,t)$. Doing so, we obtain a system of two coupled equations

$$\begin{aligned} W_u(x,t)= & {} \frac{\displaystyle \kappa }{\displaystyle 2\omega } {\mathcal {K}} H_n\bigg ( {\mathcal {U}}\bigg ( w_{\textrm{ee}} W_u(x,t) - w_{\textrm{ei}}W_v(x,t), \nonumber \\{} & {} \left. \left. \frac{\displaystyle \eta _0 + \epsilon \sin {x} + i \gamma }{\displaystyle 2\omega }, \omega \right) \right) , \\ W_u(x,t)= & {} \frac{\displaystyle \kappa }{\displaystyle 2\omega } {\mathcal {K}} H_n\bigg ( {\mathcal {U}}\bigg ( w_{\textrm{ie}} W_u(x,t) - w_{\textrm{ii}}W_v(x,t), \nonumber \\{} & {} \left. \left. \frac{\displaystyle \eta _v + i \gamma }{\displaystyle 2\omega }, \omega \right) \right) . \end{aligned}$$

One difference between this model and the ones studied above is that the solution cannot be shifted by a constant amount in *x* to ensure that it is always even (or odd) about a particular point in the domain. Thus we need to write

37 $$\begin{aligned} W_u(x,t)= & {} \sum \limits _{m=0}^{2F} ( v_m^u + w_m^u \sin x +z_m^u \cos x ) \psi _m(t) \end{aligned}$$

38 $$\begin{aligned} W_v(x,t)= & {} \sum \limits _{m=0}^{2F} ( v_m^v + w_m^v \sin x +z_m^v \cos x ) \psi _m(t) \end{aligned}$$

These equations contain $6(2F+1)$ unknowns and we find them (and $\omega$) in the same way as above, by projecting the self-consistency equations for $W_u(x,t)$ and $W_v(x,t)$ onto the different spatio-temporal Fourier modes to obtain equations similar to (15)–(16).

**Fig. 9:**
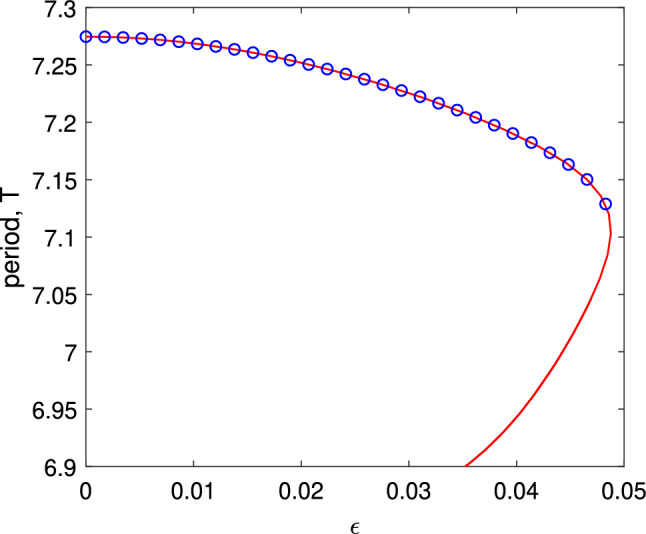
Period, *T*, of a modulated travelling wave solution of Eqs. (31)–(32) as a function of heterogeneity strength $\epsilon$. Circles are from direct simulation of Eqs. (31)–(32). Other parameters are as in Fig. 8

The results of varying the heterogeneity strength $\epsilon$ are shown in Fig. 9. Increasing heterogeneity decreases the period of oscillation, and eventually the travelling wave appears to be destroyed in a saddle-node bifurcation.

We conclude this section by noting that for some parameter values the model (31)–(32) can show periodic solutions which do not travel, like those shown in Sect. 2. Likewise, the model in Sect. 1 can support travelling waves for $\kappa =2$.

### Winfree oscillators

One of the first models of interacting oscillators studied is the Winfree model (Ariaratnam and Strogatz 2001; Laing et al. 2021; Pazó and Montbrió 2014; Gallego et al. 2017). We consider a spatially-extended network of Winfree oscillators whose dynamics are given by

$$\begin{aligned} \frac{\displaystyle \textrm{d}\theta _j}{\displaystyle \textrm{d}t}=\omega _j+\epsilon \frac{2\pi Q(\theta _j)}{N}\sum _{k=1}^N K_{jk}P(\theta _k) \end{aligned}$$

where $K_{jk}=K(2\pi |j-k|/N)$ for some $2\pi$-periodic coupling function *K*, $Q(\theta )=-\sin {\theta }/\sqrt{\pi }$ and $P(\theta )=(2/3)(1+\cos {\theta })^2$ is a pulsatile function with its peak at $\theta =0$. The $\omega _j$ are randomly chosen from a Lorentzian with centre $\omega _0$ and width $\Delta$ and $\epsilon$ is the overall coupling strength.

In the limit $N\rightarrow \infty$, using the Ott/Antonsen ansatz, one finds that the network is described by the equation (Laing 2017)

39 $$\begin{aligned} \frac{\displaystyle \partial z}{\displaystyle \partial t}=\frac{\epsilon }{2\sqrt{\pi }} {\mathcal {K}} \widehat{H}(z) +(i\omega _0-\Delta )z-\frac{\epsilon }{2\sqrt{\pi }} z^2 {\mathcal {K}} \widehat{H}(z), \end{aligned}$$

where the integral operator ${\mathcal {K}}$ is again defined by (5) and

$$\begin{aligned} \widehat{H}(z)=(2/3)[3/2+z+\bar{z}+(z^2+\bar{z}^2)/4]. \end{aligned}$$

**Fig. 10:**
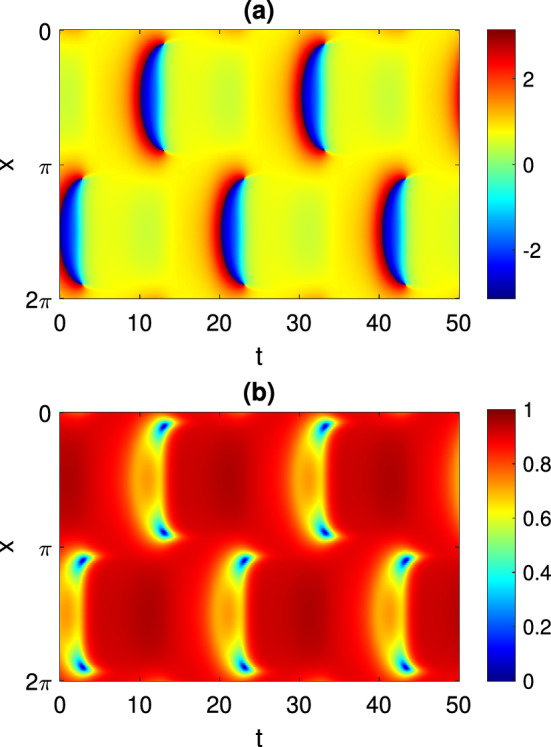
A periodic solution of Eq. (39). **a** $\arg {(z(x,t))}$. **b** |*z*(*x*, *t*)|. Parameters: $\omega _0=1$, $\Delta =0.1$, $\epsilon =2.1$

A typical periodic solution of Eq. (39) for the choice

$$\begin{aligned} K(x)=0.1+0.3\cos {x}, \end{aligned}$$

is shown in Fig. 10.

If we rescale time by the frequency of periodic solution $\omega >0$, defining $u(x,t) = z(x,t/\omega )$, and denote

$$\begin{aligned} W(x,t) = \frac{\epsilon }{2\sqrt{\pi }\omega } {\mathcal {K}} \widehat{H}(u), \end{aligned}$$

then Eq. (39) can be recast as a complex Riccati equation

40 $$\begin{aligned} \frac{\displaystyle \partial u}{\displaystyle \partial t} = W(x,t) + i \frac{\displaystyle \omega _0 + i \Delta }{\displaystyle \omega } u - W(x,t) u^2. \end{aligned}$$

From the Proposition 2 in Omel’chenko (2023), it follows that for every $\omega ,\Delta > 0$, $\omega _0\in {\mathbb {R}}$ and for every real-valued, $2\pi$-periodic in *t* function *W*(*x*, *t*), Eq. (40) has a $2\pi$-periodic solution lying in the open unit disc ${\mathbb {D}}$. Denoting the corresponding solution operator

$$\begin{aligned} \widehat{{\mathcal {U}}}: C_{\textrm{per}}([0,2\pi ];{\mathbb {R}})\times {\mathbb {C}}_{\textrm{up}}\rightarrow C_{\textrm{per}}([0,2\pi ];{\mathbb {D}}), \end{aligned}$$

we easily obtain a self-consistency equation for periodic solutions of Eq. (40)

41 $$\begin{aligned} W(x,t) = \frac{\epsilon }{2\sqrt{\pi }\omega } {\mathcal {K}} \widehat{H}\left( \widehat{{\mathcal {U}}}\left( W(x,t), \frac{\displaystyle \omega _0 + i \Delta }{\displaystyle \omega } \right) \right) . \end{aligned}$$

Since the Poincaré map of Eq. (40) coincides with the Möbius transformation, we can again use the calculation scheme of Sect. 2.1 to find the value of operator $\widehat{{\mathcal {U}}}$. Thus we can solve Eq. (41) numerically for the real-valued field *W*(*x*, *t*) and frequency $\omega$.

**Fig. 11:**
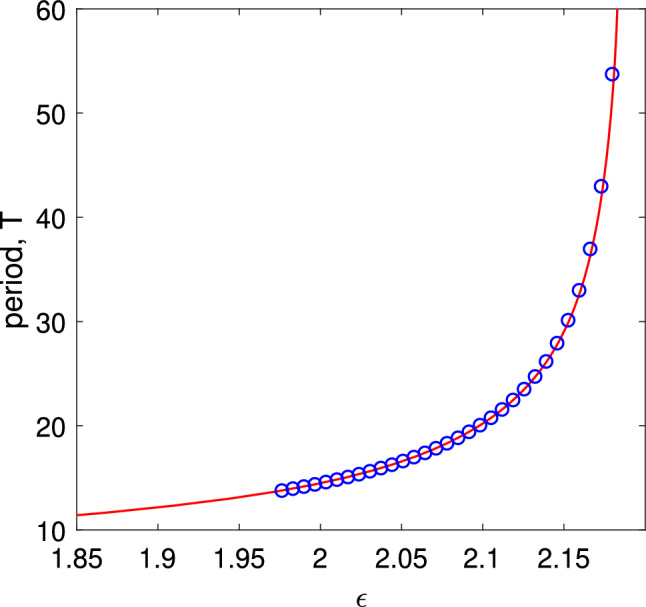
Period, *T*, of periodic solutions of Eq. (39) of the form shown in Fig. 10. Circles are from direct simulations of Eq. (39). Parameters: $\omega _0=1$, $\Delta =0.1$

Following the periodic solution shown in Fig. 10 as $\epsilon$ is varied we obtain Fig. 11. As shown by the circles (from direct simulations of Eq. (39)) this solution is not always stable. As $\epsilon$ is decreased the solution loses stability to a uniformly travelling wave, and the period of this wave is not plotted.

## Discussion

We considered time-periodic solutions of the Eqs. (4)–(5), which exactly describe the asymptotic dynamics of the network (1) in the limit of $N\rightarrow \infty$. At every point in space, Eq. (4) is a Riccati equation and we used this to derive a self-consistency equation that every periodic solution of Eq. (4) must satisfy. The Poincaré map of the Riccati equation is a Möbius map and we can determine this map at every point in space using just three numerical solutions of the Riccati equation. Knowing the Möbius map enables us to numerically solve the self-consistency equation in a computationally efficient manner. We showed the results of numerically continuing several types of periodic solutions as a parameter was varied.

We derived equations governing the stability of such periodic solutions, but solving these equations is numerically challenging. We also derived the expression for the mean firing rate of neurons in a network in terms of the quantities already calculated in the self-consistency equation. We finished in Sect. 3 by demonstrating the application of our approach to several other models involving delays, two populations of neurons, and a network of Winfree oscillators.

Our approach relies critically on the mathematical form of the continuum-level equations (they can be written as a Riccati equation) which are derived using the Ott/Antonsen ansatz, valid only for phase oscillators whose dynamics and coupling involve sinusoidal functions of phases or phase differences. Other systems for which our approach should be applicable include two-dimensional networks which support moving or “breathing” solutions (Bataille-Gonzalez et al. 2021); however the coupling function would have to be of the form such that the integral equivalent to (5) could be written exactly using a small set of spatial basis functions. Another application would be to any system which is periodically forced in time and responds in a periodic way (Segneri et al. 2020; Reyner-Parra and Huguet 2022; Schmidt et al. 2018).

## Data Availability

Code for calculating any of the results presented here is available on reasonable request from the authors.

## Appendix

Let us consider a complex Riccati equation

42 $$\begin{aligned} \frac{\displaystyle \textrm{d}z}{\displaystyle \textrm{d}t} = c_0(t) + c_1(t) z + c_2(t) z^2 \end{aligned}$$

with $2\pi$-periodic complex-valued coefficients $c_0(t)$, $c_1(t)$ and $c_2(t)$. In this section we prove a statement which is a modified version of Proposition 3 from Omel’chenko (2023).

### Proposition 2

Suppose that there is $c_* > 0$ such that

43 $$\begin{aligned} \textrm{Re}\,( \overline{c_0(t)} z + c_1(t) + c_2(t) z ) \le - c_* \textrm{Re}\,(z + 1) \end{aligned}$$

for all $z\in \overline{{\mathbb {D}}}$ and $0\le t \le 2\pi$, and that $z = - 1$ is not a fixed point of Eq. (42). Then, the Poincaré map of Eq. (42) is described by a hyperbolic or loxodromic Möbius transformation. Moreover, the stable fixed point of this map lies in the open unit disc ${\mathbb {D}}$, while the unstable fixed point lies in the complementary domain $\hat{{\mathbb {C}}}\backslash \overline{{\mathbb {D}}}$, where $\hat{{\mathbb {C}}} = {\mathbb {C}}\cup \{\infty \}$ is the extended complex plane. For Eq. (42) this means that it has exactly one stable $2\pi$-periodic solution and this solution satisfies $|z(t)| < 1$ for all $0\le t \le 2\pi$.

### Proof

The fact that the Poincaré map of Eq. (42) is described by a Möbius transformation was shown elsewhere (Campos 1997; Wilczyński 2008). So we only need to show that such a Möbius transformation ${\mathcal {M}}(z)$ maps all points of the unit circle $|z| = 1$ into the open unit disc ${\mathbb {D}}$ (but not on its boundary). Then we can repeat the arguments of the proof of Proposition 2 from Omel’chenko (2023).

Suppose the opposite. Then Eq. (42) has a solution $z_*(t)$ such that $|z_*(0)| = |z_*(2\pi )| = 1$. Due to the inequality (43) and the Proposition 1 from Omel’chenko (2023) this solution satisfies $|z_*(t)| \le 1$ for all $t\in [0,2\pi ]$. Moreover, since $z = -1$ is not a fixed point of Eq. (42), we can always choose $t_*\in (0,2\pi )$ such that $z_*(t)\ne -1$ for $t\in [t_*,2\pi )$. Then the Mean Value Theorem implies

44 $$\begin{aligned} 0 \le |z_*(2\pi )|^2 - |z_*(t_*)|^2 = 2 \pi \frac{\displaystyle \textrm{d}|z_*|^2}{\displaystyle \textrm{d}t}(t_{**}) \end{aligned}$$

for some $$t_{**}\in (t_*,2\pi )$$. On the other hand, due to our assumptions, we have

$$\begin{aligned} \frac{\displaystyle \textrm{d}|z_*|^2}{\displaystyle \textrm{d}t}= & {} 2 \textrm{Re}\,\left( \overline{c_0(t)} z_*(t) + c_1(t) + c_2(t) z_*(t) \right) \\ {}\le & {} - 2 c_* \textrm{Re}\,(z_*(t) + 1) \end{aligned}$$

and therefore

$$\begin{aligned} \frac{\displaystyle \textrm{d}|z_*|^2}{\displaystyle \textrm{d}t}(t_{**}) \le - 2 c_* \textrm{Re}\,(z_*(t_{**}) + 1) < 0. \end{aligned}$$

This is a contradiction to (44), which completes the proof.  $\square$

### Remark 1

If the conditions of Proposition 2 are fulfilled, then the stable solution of Eq. (42) is also asymptotically stable. This result follows from the properties of stable fixed points of hyperbolic and loxodromic Möbius transformations.

### Remark 2

For every fixed *x* Eq. (7) from the main text is equivalent to Eq. (42) with

$$\begin{aligned} c_0(t) = c_2(t) = i \left( W(x,t) + \zeta - \frac{\displaystyle 1}{\displaystyle 2\omega } \right) \end{aligned}$$

and

$$\begin{aligned} c_1(t) = 2 i \left( W(x,t) + \zeta + \frac{\displaystyle 1}{\displaystyle 2\omega } \right) , \end{aligned}$$

where *W*(*x*, *t*) is a real-valued function, $\omega$ is a positive constant, and $\zeta = (\eta _0 + i \gamma ) / (2\omega )$ with $\eta _0\in {\mathbb {R}}$ and $\gamma > 0$. In this case, we have

$$\begin{aligned} \textrm{Re}\,( \overline{c_0(t)} z + c_1(t) + c_2(t) z ) = - \frac{\displaystyle \gamma }{\displaystyle \omega } \textrm{Re}\,(z + 1) \end{aligned}$$

and therefore inequality (43) is satisfied. On the other hand, we have

$$\begin{aligned} c_0(t) + c_1(t) (-1) + c_2(t) (-1)^2 = - 2 i / \omega \ne 0 \end{aligned}$$

and therefore $z = -1$ is not a fixed point of the corresponding Riccati equation. Thus, all of the conclusions of Proposition 2 hold true for Eq. (7).

### Remark 3

If the conditions of Proposition 2 are fulfilled, then the stable solution of Eq. (42) can be computed in the following way.

(i) One solves Eq. (42) on the interval $t\in (0,2\pi ]$ with three different initial conditions $z(0) = z_k\in {\mathbb {D}}$, $k=1,2,3$, and obtains three solutions $Z_k(t)$. Since each $z_k$ lies in the open unit disc ${\mathbb {D}}$ this automatically implies $|Z_k(t)| < 1$ for all $t\in (0,2\pi ]$.

(ii) One denotes $w_k = Z_k(2\pi )$. Then, due to the properties of Poincaré map one has $w_k = {\mathcal {M}}(z_k)$, $k=1,2,3$, where ${\mathcal {M}}(z)$ is a Möbius transformation representing this map. The above three relations can be used to reconstruct the map ${\mathcal {M}}(z)$, namely

$$\begin{aligned} {\mathcal {M}}(z) = \frac{a z + b}{c z + d} \end{aligned}$$

where

$$\begin{aligned} a= & {} \det \left( \begin{array}{ccc} z_1 w_1 &{} w_1 &{} 1 \\ z_2 w_2 &{} w_2 &{} 1 \\ z_3 w_3 &{} w_3 &{} 1 \end{array} \right) , \quad b = \det \left( \begin{array}{ccc} z_1 w_1 &{} z_1 &{} w_1 \\ z_2 w_2 &{} z_2 &{} w_2 \\ z_3 w_3 &{} z_3 &{} w_3 \end{array} \right) , \\ c= & {} \det \left( \begin{array}{ccc} z_1 &{} w_1 &{} 1 \\ z_2 &{} w_2 &{} 1 \\ z_3 &{} w_3 &{} 1 \end{array} \right) , \quad d = \det \left( \begin{array}{ccc} z_1 w_1 &{} z_1 &{} 1 \\ z_2 w_2 &{} z_2 &{} 1 \\ z_3 w_3 &{} z_3 &{} 1 \end{array} \right) . \end{aligned}$$

(iii) Once the map ${\mathcal {M}}(z)$ is known, one can find its fixed points by solving the quadratic equation

$$\begin{aligned} c z^2 + d z - a z - b = 0. \end{aligned}$$

This yields two roots

$$\begin{aligned} z_\pm = \frac{\displaystyle a - d \pm \sqrt{ (a - d)^2 + 4 b c }}{\displaystyle 2 c}. \end{aligned}$$

(iv) Choosing from the roots $z_+$ and $z_-$ the one that lies in the unit disc ${\mathbb {D}}$, one obtains the initial condition that determines the periodic solution of interest. The latter can be computed by solving Eq. (42) with this initial condition.

(v) Sometime it may happen that the Poincaré map ${\mathcal {M}}(z)$ is strongly contracting so that

$$\begin{aligned} |w_1 - w_2 | + |w_3 - w_2 | < 10^{-8}, \end{aligned}$$

where the value $10^{-8}$ is chosen through experience. In this case, the calculations in steps (ii) and (iii) become inaccurate. Then the initial condition of the periodic solution of interest is approximately given by the average $(w_1 + w_2 + w_3) /3$.

The above steps (i)–(v) can be understood as a constructive definition of the solution operator of Eq. (42), which for every admissible choice of $2\pi$-periodic coefficients $c_1(t)$, $c_2(t)$ and $c_3(t)$ yields the corresponding stable $2\pi$-periodic solution of Eq. (42). More detailed justification of this definition can be found in Omel’chenko (2023, Section 4). The algorithm in Sect. 2.1 consists of applying the procedure above at every point on the spatial grid (in parallel).
